# Sources of error in emergency ultrasonography

**DOI:** 10.1186/2036-7902-5-S1-S1

**Published:** 2013-07-15

**Authors:** Antonio Pinto, Fabio Pinto, Angela Faggian, Giuseppe Rubini, Ferdinando Caranci, Luca Macarini, Eugenio Annibale Genovese, Luca Brunese

**Affiliations:** 1 Department of Diagnostic Imaging, A. Cardarelli Hospital, Naples, Italy; 2 Department of Diagnostic Imaging, Marcianise Hospital, ASL Caserta (CE), Italy; 3 Department of Internal and Experimental Medicine, Magrassi-Lanzara, Institute of Radiology, Second University of Naples, Naples, Italy; 4 Nuclear Medicine Unit, Bari, Italy; 5 Department of Radiology and Radiotherapy, University of Naples Federico II, Naples, Italy; 6 Department of Radiology, University of Foggia, Foggia, Italy; 7 Department of Radiology, University of Cagliari, Cagliari, Italy; 8 Department of Health Science, University of Molise, Campobasso, Italy

**Keywords:** Emergency Ultrasonography, Error, Malpractice, Medical negligence

## Abstract

**Background:**

To evaluate the common sources of diagnostic errors in emergency ultrasonography.

**Methods:**

The authors performed a Medline search using PubMed (National Library of Medicine, Bethesda, Maryland) for original research and review publications examining the common sources of errors in diagnosis with specific reference to emergency ultrasonography. The search design utilized different association of the following terms : (1) emergency ultrasonography, (2) error, (3) malpractice and (4) medical negligence. This review was restricted to human studies and to English-language literature. Four authors reviewed all the titles and subsequent the abstract of 171 articles that appeared appropriate. Other articles were recognized by reviewing the reference lists of significant papers. Finally, the full text of 48 selected articles was reviewed.

**Results:**

Several studies indicate that the etiology of error in emergency ultrasonography is multi-factorial. Common sources of error in emergency ultrasonography are: lack of attention to the clinical history and examination, lack of communication with the patient, lack of knowledge of the technical equipment, use of inappropriate probes, inadequate optimization of the images, failure of perception, lack of knowledge of the possible differential diagnoses, over-estimation of one’s own skill, failure to suggest further ultrasound examinations or other imaging techniques.

**Conclusions:**

To reduce errors in interpretation of ultrasonographic findings, the sonographer needs to be aware of the limitations of ultrasonography in the emergency setting, and the similarities in the appearances of various physiological and pathological processes. Adequate clinical informations are essential. Diagnostic errors should be considered not as signs of failure, but as learning opportunities.

## Background

Error in medicine has become headline news in recent years. Within radiology, the notable progress in proving disease has left error evaluation a topic infrequently explicitly explorer. The job of diagnostic radiology comprises the identification of all abnormalities in an imaging examination and their correct diagnosis [1].

The Institute of Medicine defines error as “the failure of a planned action to be completed as intended or the use of a wrong plan to achieve an aim” and estimates that between 44,000 and 98,000 Americans die each year because of medical errors [2].

Error, as defined by Stedman’s Medical Dictionary, is “a defect in structure or function. A mistaken decision”[3]. Wu and colleagues [4] define medical error as “a commission or an omission with potentially negative consequences for the patient that would have been judged wrong by skilled and knowledgeable peers at the time it occurred, independent of whether there were any negative consequences.”

Errors in medical imaging have been observed since the early period of radiology, as reported by Garland [5] in 1959. The “surprising” degree of errors first reported over 50 years ago seem to have persisted and unchanged. Some of the techniques are particularly vulnerable to errors: these include chest X-rays, with a “miss rate” of 20-50% [6], and mammography, with a “miss rate” of up to 75% [7].

Ultrasonography (US) has become an important diagnostic tool for an increasing number and range of clinical conditions, such as the detection of abdominal masses or as the first procedure used in the evaluation of trauma or non-traumatic acute abdominal conditions.

Emergency US is particularly susceptible to errors, more than any other diagnostic imaging technique: in fact, the misinterpretation of sonographic images should be considered as a serious risk in US-based diagnosis [8].

In this article we evaluate the common sources of diagnostic errors in emergency ultrasonography through a literature search.

## Methods

The authors performed a Medline search using PubMed (National Library of Medicine, Bethesda, Maryland) for original research and review publications examining the common sources of errors in diagnosis with specific reference to emergency ultrasonography. Systematic literature review was performed from 1990 to 2013. The search design utilized different association of the following terms : (1) emergency ultrasonography, (2) error, (3) malpractice and (4) medical negligence. This review was restricted to human studies and to English-language literature. Four authors reviewed all the titles and subsequent the abstract of 171 articles that appeared appropriate. The abstracts were reviewed and selected based on well-designed methodology, diagnostic accuracy and outcomes.

Additional articles were recognized by reviewing the reference lists of relevant papers. Finally, the full text of 48 selected articles was reviewed.

## Results

### Causes of error in emergency ultrasonography

Causes of error in emergency ultrasonography are multifactorial, frequently exist in combination as in other diagnostic imaging techniques [9,10] and include: lack of attention to the clinical history and examination, lack of communication with the patient (who may be uncooperative), lack of knowledge of the technical equipment, use of inappropriate probes, inadequate optimization of the images, failure of perception, lack of knowledge of the possible differential diagnoses, over-estimation of one’s own skill, failure to suggest further ultrasound examinations or other imaging techniques (such as CT or MRI) [11-16].

### Error in ultrasonographic technique

In clinical practice, correct choices regarding the transducer, the setting of the technical equipment, and the amount of sonographic gel are fundamental to obtain usable diagnostic images.

The proper functioning of the ultrasound transducer is a key factor for reliable diagnosis by ultrasound. This function depends highly on the condition of the piezoelectric elements and on the wires within the transducer. It is also important that the function of the matching layers in front of the elements and the backing material behind the elements work properly [17]. Modern US equipment is pre-loaded with “pre-sets”, a set of pre-determined parameters related to the different organs and type of patient. While such pre-sets are useful as screening images or as an initial approach, they almost always need to be changed on the basis of the clinical scenario and disease [15].

Another source of misinterpretation and error in ultrasonography concerns artifacts: image artifacts are frequenlty encountered in clinical ultrasonography and may be a cause of confusion for the sonographer. Some artifacts may be avoidable and arise secondary to improper scanning technique. Other artifacts are generated by the physical limitations of the modality. US artifacts arise secondary to errors inherent to the ultrasound beam characteristics, the presence of multiple echo paths, velocity errors, and attenuation errors. The ability to recognize and remedy potentially correctable US artifacts is important for image quality improvement and optimal patient care [18].

### Technical skill of the operator

An important source of error in emergency ultrasonography depends on the technical skill of the operator: a correct ultrasonographic examination is directly related to operator skill, training, and experience. The sonographer’s responsibilities include maximal benefit of the diagnostic capability of ultrasonography, the knowledge of what to look for, and the competence to interpret the ultrasonographic findings based on the understanding of the physiology and pathological changes of the examined organs.

Modern ultrasound equipment is certainly adequate for producing images that permit diagnosis of anomalies such as open lumbosacral spina bifida or atrioventricular septal defect. However, such diagnoses can only be made if considerable operator skill is associated with knowledge and experience.

### Errors in obstetric and gynecologic ultrasound

The earliest litigation related to diagnostic ultrasound occurred in 1974 and involved obstetric measurements. Before 1974, images were so difficult to interpret that ultrasonography was considered of little value apart from obstetric measurement data and for characterizing masses as cysts [19]. Performing obstetric sonography brings serious medico- legal risk [20], because overlooking a detectable fetal abnormality often results in the largest indemnification payments in medical malpractice [21,22]. Whether sonographers perform the examination themselves or rely on a technologist to achieve the sonographic images, it is the sonographer who is responsible for the quality of the examination [23,24]. The sonographer must make sure that basic anatomy is depicted in a pertinent manner and that all measurements are accurate [25,26]. Pregnancy ultrasound examinations should include a complete structural survey to avoid missing fetal anomalies: if the sonographic examination is suboptimal and therefore has to be repeated, the second sonographic examination should be repeated in its entirety. The attending physician should be called if significant abnormalities or fetal anomalies are suspected.

General radiologists who miss subtle fetal abnormalities on sonography and claim malpractice immunity because they are not “sonographic specialists” cannot escape liability any more than those who miss a subarachnoid hemorrhage on a CT scan and claim malpractice immunity because they are not neuroradiologists.

### Errors in ultrasonography the emergency room setting

The emergency room setting presents a scenario ripe for malpractice claims. Quick diagnosis and treatment of patients with whom we have had no previous contact, and who, quite often, may be uncooperative, and/or under the influence of alcohol or drugs creates an environment with significant risk [27]. The frequency of reported “missed diagnoses” depends on how the frequency of error was assessed: based on trauma registries, error rates were approximately 2% [28], while retrospective chart review found approximately 40% [29], and retrospective review of all admissions revealed missed or delayed diagnoses of approximately 8%-10% [28-30].

In the management of traumatized patients, an error can increase the rate of mortality and morbidity: most diagnostic errors (downgrading of an injury) in radiology occur using traditional imaging studies, i.e., plain film and ultrasound, because of their intrinsic low resolution and/or limited field of view. Ultrasonography is highly operator-dependent, so it is crucial that the sonographer is accurately trained in order to be able to implement the diagnostic capabilities of ultrasonography [15]. As a first step in proper US scanning, the sonographer must be aware of the circumstances of the injury, the patient’s symptoms, and the clinical findings [31,32]. Moreover, the sonographer should evaluate the patient in terms of physical constitution (in obese patients, the thickness of subcutaneous fat and the sound-attenuating properties of fat present challenges) and the presence of conditions potentially limiting the examination (such as obliged decubitus, scars, etc.). The sonographer should be aware of the limitations of the technique in the evaluation of the traumatized patients, asking for other diagnostic imaging procedures (Multidetector row Computed Tomography). On the other hand, the use of Multidetector row Computed Tomography in trauma patients requires the adoption of tailored protocols and skill to highlight subtle or even minimal signs of injuries [33-37].

## Discussion

Medical errors represent a serious public health problem and pose a threat to patient safety. Error is inevitable in medicine. Errors are common in radiological diagnosis. They can arise during acquisition of images, processing, and interpretation [38-40].

Radiologists performing US in the emergency setting cover a large range of ultrasonographic examinations from abdominal to vascular: this exposes interpreters to a wider variety of possible errors or at least perceived errors. For instance, the testicular US examination to rule out torsion may lead to organ loss and litigation if torsion is missed and cases are common.

Emergency ultrasonography is particularly susceptible to errors, more than any other diagnostic imaging technique: acquisition of accurate ultrasonographic images depends on the operator. The correct choice of ultrasound transducers, ultrasound frequency, and ultrasonographical skills are essential in reducing errors during acquisition.

In gynaecological ultrasonography, most of the problems relating to acquisition of correct images of the pelvic organs may be overcome with the use of transvaginal scanning. Ultrasound image processing depends on a number of physical factors of ultrasound itself and its interactions with body structures [41].

Litigation related to diagnostic ultrasound has become progressively more frequent as images have become easier to interpret, expectations inherent to the capacity of diagnostic ultrasound to facilitate diagnoses of subtle fetal anomalies have become higher, and sonographic equipment has become more widespread. Obstetric ultrasound has always attracted more litigation than other aspects of diagnostic ultrasound. There has been a change in the main target of litigation over time: in the 1980s, ectopic pregnancy was the most common reason for litigation; today, litigation related to a missed fetal anomaly is the most frequent indication [19].

Types of litigation in ultrasonography (including the ultrasonography performed in the emergency setting) involves the following groups: missed diagnoses, misinterpreted sonograms, invented lesions, delay in communicating information to a clinician, failure to perform sonography, fraud cases, procedure-related cases, and sonographer-related suits [19].

Errors in emergency ultrasonography can be reduced by improvements both in knowledge and in systems. Improved knowledge and skills may include: awareness of history and clinical symptoms, comparison with previous studies, careful selection of the initial and subsequent radiological or clinical investigation [42]. Systems changes include: improvement in working conditions and in the time available for reporting, equipment alteration to prevent accidental error, and regular dialogue in the emergency room between clinicians and sonographers [42].

An important goal of error analysis is to create processed aimed at reducing or preventing the occurrence of errors and minimizing the degree of harm [43,44]. The science of measuring diagnostic errors is underdeveloped [45,46] and the implementation of a peer review process in diagnostic radiology is one method of responding to this need.

Educational programs, morbidity meetings and a comprehensive and respected root cause analysis process are important for decreasing the likelihood of future diagnostic errors.

The urgent need to raise the level of ultrasound education worldwide has been recognized by the World Health Organization. An impoverished nation cannot afford to invest heavily in expensive, highly technological diagnostic imaging equipment. Ultrasound scanners, however, are relatively inexpensive and highly effective in the hands of a trained operator. More importantly, ultrasound is a “sustainable technology” for developing and impoverished nations because of its relatively low cost of purchase, low cost for maintenance and supplies, portability, and durability in comparison with all other imaging modalities [47]. Moreover, early education of operators is a priority that can begin to be addressed in medical school. The practice of ultrasound has clearly been shown to be operator-dependent, and the way to train better operators is to start early, provide opportunities for practice, and standardize curriculum that will ultimately align with residency requirements in the various specialties [48].

## Limitations

There are several limitations to our study. Some relevant to this review include: accuracy, unobtainable texts and timeframe restrictions. In this review we do not used statistical techniques for combining results of the eligible studies. Moreover it is possible that the type and distribution of errors in emergency ultrasonography would be different in other clinical settings.

## Conclusions

The main reason for studying medical errors is to try to prevent them. Errors in emergency ultrasonography fall into recurrent patterns. The discovery of any errors presents an opportunity to study the types that occur and to examine their sources and develop interventions to prevent them from recurring. To reduce errors in interpretation of ultrasonographic findings, the sonographer needs to be aware of the limitations of ultrasonography in the emergency setting, and the similarities in the appearances of various physiological and pathological processes. Adequate clinical informations are essential.

Confirmation of findings with other imaging modality such as MDCT or MRI, when appropriate, may be required. More openness about the incidence of error in emergency ultrasonography, as well as departmental practice of peer support instead of blame, could help sonographers learn from mistakes and improve their performance.

## Competing interests

The authors declare that they have no competing interests.

## Authors’ contributions

AP participated in the conception, design, and coordination of the study as well as in the acquisition and analysis of the data and helped draft the manuscript. FP participated in the acquisition and analysis of the data as well as in the drafting and revision of the manuscript for submission. AF, GR, FC, LM, EAG, participated in the conception and design of the study and in the revision of the manuscript. LB participated in the acquisition and analysis of the data as well as in the drafting and revision of the manuscript for submission.

All authors read and approved the final manuscript.

## Declarations

This article has been published as part of *Critical Ultrasound Journal* Volume 5 Supplement 1, 2013: Topics in emergency abdominal ultrasonography. The full contents of the supplement are available online at http://www.criticalultrasoundjournal.com/supplements/5/S1. Publication of this supplement has been funded by the University of Molise, University of Siena, University of Cagliari, University of Ferrara and University of Turin.
